# Significantly reduced incidence and improved survival from prostate cancer over 25 years

**DOI:** 10.1186/s12889-023-17440-7

**Published:** 2023-12-21

**Authors:** Bernat Carles Serdà-Ferrer, Arantza Sanvisens, Rafael Fuentes-Raspall, Montse Puigdemont, Xavier Farré, Anna Vidal-Vila, Martí Rispau-Pagès, Alicia Baltasar-Bagué, Rafael Marcos-Gragera

**Affiliations:** 1 Department of Nursing, Universitat de Girona, 17003 Girona, Spain; 2 Epidemiology Unit and Girona Cancer Registry, Institut Català d’Oncologia, Pla Director d’Oncologia, Institut d’Investigació Biomèdica de Girona Dr. Josep Trueta (IDIBGI), 17004 Girona, Spain; 3 Radiation Oncology Department, Institut Català d’Oncologia, Institut d’Investigació Biomèdica de Girona Dr. Josep Trueta (IDIBGI), 17007 Girona, Spain; 4 Department of Health, Agència de Salut Pública de Catalunya, 25006 Lleida, Spain; 5 Registre de Tumors Hospitalari (RTH ICO-ICS), Institut Català d’Oncologia, Hospital Universitari Dr. Josep Trueta, Institut d’Investigació Biomèdica de Girona Dr. Josep Trueta (IDIBGI), 17007 Girona, Spain

**Keywords:** Prostate cancer, Incidence, Mortality, Epidemiology, Survival, Temporal trends

## Abstract

**Background:**

Prostate cancer (PCa) was the second most frequent cancer and the fifth leading cause of cancer death among men in 2020. The aim of this study was to analyze trends in the incidence, mortality and survival of PCa in Girona, Spain, over 25 years.

**Methods:**

Population-based study of PCa collected in the Girona Cancer Registry, 1994–2018. Age-adjusted incidence and mortality rates were calculated per 100,000 men-year. Joinpoint regression models were used for trends, calculating the annual percentage changes (APC). Observed and net survival were analyzed using Kaplan–Meier and Pohar-Perme estimations, respectively.

**Results:**

A total of 9,846 cases of PCa were registered between 1994–2018. The age-adjusted incidence and mortality rates were 154.7 (95%CI: 151.7 157.8) and 38.9 (95%CI: 37.3 –40.6), respectively. An increased incidence of 6.2% was observed from 1994 to 2003 (95%CI: 4.4 –8.1), and a decrease of -2.7% (95%CI: -3.5 –;-1.9) between 2003 and 2018. Mortality APC was -2.6% (95%CI: -3.3 –-2.0). Five-year observed and net survival were 72.8% (95%CI: 71.8 – 73.7) and 87.2% (95%CI: 85.9 – 88.4), respectively. Five-year net survival increased over time from 72.9% (1994–1998) to 91.3% (2014–2018).

**Conclusions:**

The analyses show a clear reduction in PCa incidence rates from 2003 on, along with an increase in overall survival when comparing the earlier period with more recent years.

**Supplementary Information:**

The online version contains supplementary material available at 10.1186/s12889-023-17440-7.

## Introduction

Prostate cancer (PCa) was the second most frequent cancer and the fifth leading cause of cancer death among men worldwide in 2020. Incidence rates vary from 6.3 to 83.4 per 100,000 men across regions, with the highest rates found in Northern and Western Europe, the Caribbean, Australia/New Zealand, Northern America, and Southern Africa, and the lowest rates in Asia and Northern Africa. Mortality rates display different patterns, ranging from 3.1 to 27.9 per 100,000 men [1].

The etiology of PCa is unknown. Established risk factors are limited to advanced age, black men, family history, and certain cancer predisposition genetic mutations (i.e., BRCA1 and BRCA2), as well as conditions such as Lynch syndrome [1]. A wide variety of individual, environmental and occupational risk factors have been proposed, including obesity, alcohol consumption, vitamin or mineral interactions, certain dietary habits, such as an excessive intake of fat, fried food or sugar-sweetened beverages, and pesticide exposure [2–5]. No initial or early symptoms are displayed in most cases. Early detection is based on the use of a prostate specific antigen (PSA), which is controversial since it leads to an overdiagnosis of indolent cancers [6]. In fact, it has been estimated that up to 35% of the PSA tests performed do not comply with the recommendations of clinical guidelines [7]. Current worldwide guidelines recommend that men receive a PSA test following a comprehensive shared decision-making process after informing them of the advantages, disadvantages and uncertainties regarding the test [8].

Incidence and mortality patterns vary widely worldwide due to differences in detection practices, availability of treatment, and underlying genetic susceptibility. PCa generally has a good prognosis and survival has been increasing in recent years. Data from the European EUROCARE-5 study showed an 8% increase in relative survival at 5 years between 1999–2001 and 2005–2007, the rate being 82% for the latter period [9]. On the other hand, the CONCORD-3 study, using data from 290 registries in 62 countries, established a 5-year survival of between 70–100% for most countries, with an increase of between 5–10% when comparing 1995–1999 and 2010–2014 in European countries such as Spain [10].

The Girona Cancer Registry (GCR) is a population-based registry that has collected information on all cancer cases in the province of Girona since 1994. After non-melanoma skin cancer, PCa is the most commonly diagnosed cancer and the third cause of mortality in men in the province of Girona [11]. PSA screening is not recommended for populational screening in Catalonia [12].

The objectives of this study are twofold: firstly, to describe trends in incidence, mortality and survival of PCa using GCR population-based data for the period 1994–2018; and secondly, to determine trends in PSA requests in the region from 2006 onwards.

## Methods

### Study population

The analysis focused on population-based data regarding invasive PCa included in the GCR during the period 1994–2018. The GCR is a population-based registry that has collected information on all cancer cases in the province of Girona, northeastern Catalonia, since 1994. It covers a population of 392,976 men (2021) and an area of 5,910 km^2^. The province of Girona is a territory linked to industry and tourism and in recent years immigration has grown to represent 20% of the population, a third of which are of African origin [13]. In Catalonia, public health care is universal, although 35% of the population has private health insurance [14].

The data of this study meet the quality controls and follow procedures and coding rules according to the standards of the International Agency for Research on Cancer (IARC) [15]. Specifically, those cases were included that had been registered with topographic code C61 according to the International Classification of Diseases for Oncology, 3rd revision (ICD-O-3).

Vital status of patients was obtained by cross-referencing data with the Mortality Registry of Catalonia, the National Death Index and a review of medical records up to December 31, 2019.

Data on deaths from PCa among people residing in Girona for the period 1994–2018 were obtained from the Mortality Registry of Catalonia, selecting those with PCa as the main cause of death according to the registered death certificate, based on codes 185 of the Classification International Diseases, 9th edition (ICD-9) for the period 1994–1998, and C61 of the ICD-10 for the years 1999–2018.

In addition, data were obtained from requests for PSA determination made in the outpatient and hospital health care centers of the public health system in the province of Girona. These data were provided by the Girona Health Area Laboratory. It should be noted that the health system in Spain is public and used by a vast majority of the population.

### Ethics statements

The methods used in this study complied with the ethical standards for medical research and the principles of good clinical practice established in the Declaration of Helsinki. The study was reviewed by the Institutional Review Board committee at the Dr. Josep Trueta Hospital Universitari de Girona (CEIM Girona, approval number 2022.023). The study did not require informed consent, according to Acts 14/1986 and 33/2011, general and relating to Spanish public health, as well as Act 8/2001 of June 14, from the 2001–2004 Statistical Plan for Catalonia, which recognizes the GCR as a statistical archive.

### Statistical analysis

Crude and age-standardized incidence and mortality rates were calculated with 95% confidence intervals (95%CI) and expressed per 100,000 men-years. Standardized incidence rates were calculated by means of the direct method, using the 2013 European reference population.

Age was stratified in 5-year intervals for standardization, and in the following groups for age-specific analysis: < 55, 55–64, 65–74, 75–84 and ≥ 85 years.

Trends for incidence and mortality rates and annual percentage changes (APCs) were analyzed and estimated using joinpoint regression models. Data derived from the GCR for patients diagnosed with PCa from January 1, 1994 through December 31, 2018, with follow-up through December 31, 2019. A total of 59 patients (0.6%) were lost to follow-up during the study period. Death certificate only (DCO) cases and those detected by autopsy were excluded from survival analyses (*n* = 199;2.0%). Observed survival (OS) and net survival (NS) were estimated overall, by 5-year intervals and by age group using the Kaplan–Meier and Pohar-Perme methods, respectively [16]. Five- and 10-year NS using the cohort approach for patients diagnosed in the three first periods were estimated; a period approach was used to calculate 5-year survival for patients diagnosed during 2014–2018, given that five years of follow-up data were not available for all patients [17].

*P* values < 0.05 were considered statistically significant. Statistical analyses were performed using Stata software (version 14.2, College Station, Texas, USA), Joinpoint Regression Program version 4.9.0.0 (Statistical Methodology and Applications Branch, Surveillance Research Program, National Cancer Institute, Bethesda, MD) and R software (version 4.0.3) [18–20].

## Results

A total of 9,846 incident cases of PCa were recorded in the GCR between 1994 and 2018. The overall mean age ± standard deviation (SD) at diagnosis was 71.5 ± 9.1 years. Table 1 shows the characteristics of the study population. Quality indicators for the GCR showed that 89.4% of included cases were microscopically verified and 2.0% of cases were reported to the registry solely on the basis of DCOs.

**Table 1:** Characteristics and incidence rates of PCa cases in Girona, 1994–2018

	**1994 – 2018** ***N*** = 9,846 ***n*** (%)
**Age at diagnosis** (years), mean ± SD	71.5 ± 9.1
< 55	337 (3.4)
55–64	1,891 (19.2)
65–74	3,897 (39.6)
75–84	2.999 (30.5)
≥ 85	722 (7.3)
**Period of diagnosis**	
1994–1998	1,250 (12.7)
1999–2003	1,958 (19.9)
2004–2008	2,259 (22.9)
2009–2013	2,274 (23.1)
2014–2018	2,105 (21.4)
**Method of diagnosis**	
Microscopic	8,802 (89.4)
Non-microscopic / Clinic	555 (5.6)
Death certificate only (DCO)	199 (2.0)
Unknown	287 (2.9)
**Deaths,** until Dec 31, 2019	5,162 (52.4)
**Crude incidence rate (95%CI)** (× 100.000 men/year)	120.2 (117.8 **–** 122.6)
**Age-adjusted incidence rate (95%CI),** (× 100.000 men/year)	154.7 (151.7 **–** 157.8)

### PCa incidence and mortality rates

**Fig. 1:**
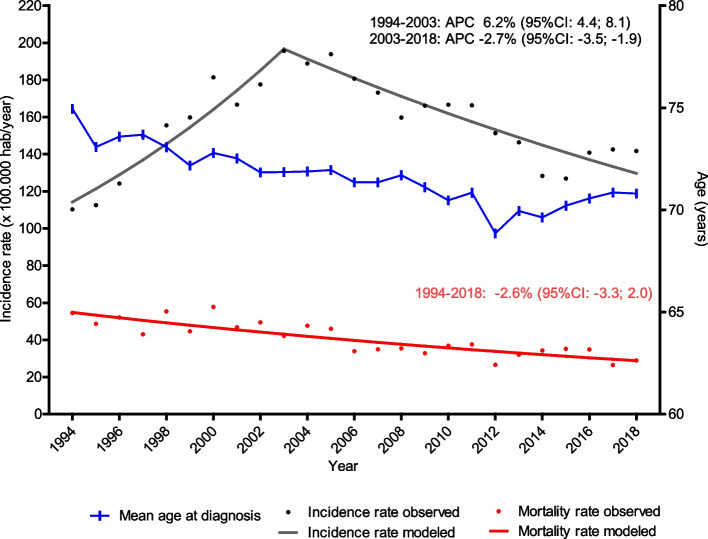
Trends in mean age of diagnosis, incidence and mortality rates of PCa in Girona, 1994–2018

The overall crude incidence rate was 120.2 cases per 100,000 men/year (95% CI: 117.8 − 122.6), while the overall age-standardized rate was 154.7 (95% CI: 151.7 − 157.8). Overall and age-adjusted incidence rates by year are shown in Supplementary Table 1. Specifically, during the period 1994–2003, the age-adjusted incidence rate gradually increased from 110.3 in 1994 to the maximum observed incidence rate of 195.7 in 2003, before subsequently decreasing to 141.7 in 2018. Figure 1 shows the application of joinpoint regression analysis to determine trends in PCa incidence during the study period observed. In this context, the APC of age-standardized incidence rate increased significantly by 6.2% (95%CI: 4.4 − 8.1; *p* < 0.001) for the first time period, while this rate declined by 2.7% (95%CI:-3.5 − -1.9; *p* < 0.001) for the second time period evaluated. Moreover, as Fig. 1 shows, a progressive decline was observed in the mean age of diagnosis throughout the evaluated period. In 1994, the mean age at diagnosis was 74.9 ± 7.6 years, while in 2018 it was 70.8 ± 8.8 years.

The age-adjusted mortality rate was 38.9 cases per 100,000 men/year (95% CI: 37.3 − 40.6) for the whole period (1994–2018). Supplementary Table 2 shows the overall and age-adjusted mortality rates by year. As displayed in Fig. 1, mortality rates decreased significantly during the period observed; in fact, the APC was -2.6% (95% CI: -3.3 − -2.0, *p* < 0.001).

### PCa survival

A total of 5,162 (52.4%) men with PCa died during follow-up. The median follow-up was 6.2 years (interquartile range: 2.6 – 10.7 years). Overall, the 5-year OS was 72.8% (95%CI: 71.8 – 73.7). The 5-year NS was 87.2% (95%CI: 85.9 – 88.4), with an increase observed over time: 72.9% (95%CI: 68.8 – 76.6), 1994–1998, 85.2% (95%CI: 82.1 – 87.8) in 1999–2003, 90.0% (95%CI: 87.3 – 92.2) in 2004–2008, 91.2% (95%CI: 88.7 – 93.2) in 2009–2013; and 91.3% (95%CI: 89.6 – 92.7) in the period 2014–2018.

**Fig. 2:**
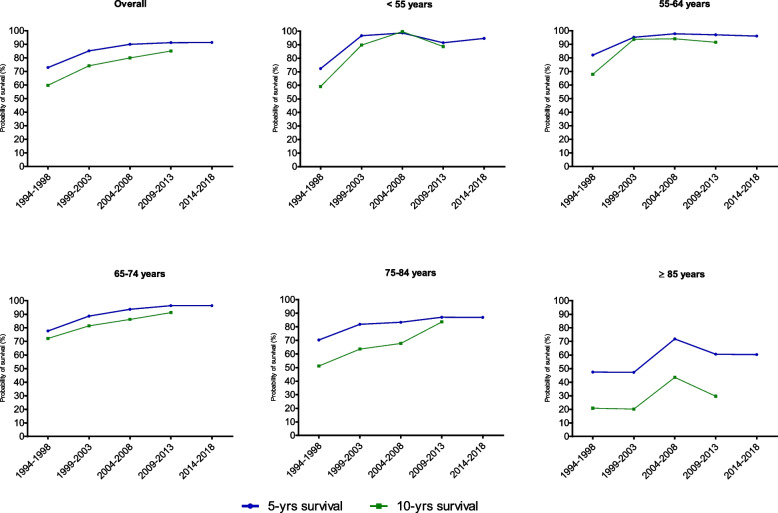
Overall and age-specific 5- and 10-year net survival of PCa in Girona by period of diagnosis

The 10-year OS was 51.7% (95%CI: 50.6 – 52.9), this being 35.8% for cases diagnosed in 1994–1998; 48.7% in 1999–2003; 54% in 2004–2008; and 59.0% in 2009–2013. The 10-year NS was 76.7% (95%CI: 74.3 – 78.9). Figure 2 shows the 5- and 10-year NS of PCa cases by age group and period of diagnosis. Supplementary Table 3 and 4 show age-specific five- and ten-year survival (95%CI), respectively.

### Data for requested PSA

Supplementary Fig. 2 shows the total number of PSA requests in the clinical environment during 2006–2018. Trend analysis showed an increase in PSA tests between 2006 and 2010, followed by a decrease through 2014 of around 2,800 tests/year, reaching stability thereafter.

## Discussion

This study describes the incidence, mortality, and survival trends in a sample of 9,846 incident cases of PCa registered over a 25 year-period. PCa is a major public health problem, being the most frequent cancer in men in the European Union, excluding non melanoma skin cancer [11, 21]. This is also true of the case studied here: the province of Girona, northeastern Spain.

The main finding of this study is the change in the trend of incidence that occurred from 2003 onwards. From 1994 to 2003, it increased significantly, with an APC of 6%; the incidence rate then decreased to 2.7% from 2003 to 2018, similarly to the trend observed in other studies for the same period [22, 23]. According to the data published by the Spanish Network of Cancer Registries (REDECAN), no decrease in incidence was observed in Spain prior to 2004 [24]. The changes in incidence trends (increasing, decreasing) could, in part, be related to screening recommendations; specifically, PSA testing was a standard screening tool for diagnosing PCa in Spain from 1993 to 2013. After this period, the recommendation was restricted to men aged between 50 and 70, with a prostate biopsy being offered to those presumed to have a high risk of cancer after a confirmatory test such as prostate MRI. In this context, PSA was used to control for disease progression after 2013 [25, 26]. An increase in the incidence of PCa in relation to the use of PSA testing from the mid-1990s has been widely described and discussed, and is associated with overdiagnosis and overtreatment. However, the subsequent decline has been more controversial; in fact, high variability has been observed in incidence rates and their trend, due in part to differing detection strategies (PSA testing) and health policies in each region [1, 27].

While it is true that the 2012 recommendations by the U.S. Preventive Service Task Force (USPSTF) against screening PCa with PSA at any age marked a turning point [28, 29], in this study, the change in the overall trend of incidence is observed a decade earlier, specifically in 2003. In this regard, as early as 2002 the USPSTF found no evidence to recommend routine PSA screening for PCa [30]. In addition, despite the probable overuse of PSA testing at that time (no specific data are available for our region), changes in clinical protocols and more accurate diagnoses thanks to the optimization of diagnostic imaging techniques such as MRI and subsequently ultrasonography-guided transrectal biopsy (TRUS) may have contributed to the decrease in biopsies, and consequently the decrease in incidence and overdiagnosis over the years [31, 32].

According to the clinical data available for Girona, there was a progressive decrease and then stabilization in PSA testing from 2010 onwards, a trend similar to other published data [33]. As noted above, the clinical use of PSA tests to diagnose PCa may have contributed to the increased incidence observed during the first period analyzed, leading to early diagnosis and probably overdiagnosis, and inflating the incidence data by increasing the number of latent cases diagnosed in asymptomatic men [22]. In this respect, the subsequent decrease in the number of PSA tests entails a loss of opportunity to detect cases in early stages [33, 34]. Specifically, a Cochrane review determined that screening with PSA increased the detection of localized tumors (relative risk (RR): 1.79; 95% CI, 1.19–2.70) and the proportion of men diagnosed with advanced disease (T3–T4, N1, M1) was significantly lower (RR: 0.80; 95% CI, 0.73–0.87) [34].

Mortality constitutes a unique descriptive statistic that is not affected by the usual overdiagnosis caused in PCa. This observational study shows a slight and continuous decline in mortality rates during the period observed, without identifying any changing trends. In Spain, a declining trend in mortality rates was observed by Etxebarria et al. [35] for the period 1995–2013, with a similar APC percentage, and by Cayuela et al. [22], identifying two different periods: APC of -3.3% for 1992–2008; and APC of -2.4% for 2008–2018. In fact, this decline has been observed across the board in developed countries, and is attributable to continuing therapeutic improvements combined with early diagnosis, as well as an improvement in the certification of cancer deaths [33, 36].

In addition to race/ethnicity (black men) and genetic predisposition, advanced age is one of the principal risk factors for PCa. In this study, the overall mean age at diagnosis was 71 years, with a gradual decrease over the period analyzed, probably related to diagnostic advances, as observed in screening programs in general, whether organized or not [37, 38].

Recently, other factors associated with PCa have been analyzed, and several studies have suggested that people with components of metabolic syndrome are more likely to develop PCa, especially those with high blood pressure [39, 40]. However, not all components of metabolic syndrome would contribute in the same way, since diabetes mellitus has been established as a protective factor [41]. In addition, diet is also thought to play its role in both PCa and metabolic syndrome [2, 42]. What is clear is that more studies are needed to analyze comorbidity and determine potential modifiable risk factors in these patients.

As for survival, 5- and 10- year trends in both OS and NS generally increased throughout the study period, regardless of age group. Differences between OS and NS, which are more marked than those observed for other tumors, especially occur in cancers diagnosed in older people, such as PCa, due to the greater risk of dying from causes other than cancer itself [43]. That being the case, a notable increase in survival is observed between the first and second study periods (1994–1998 vs. 1999–2003), as already described above and attributed especially to improvements in patient treatment and care [33, 44, 45]. Overall, the rates of NS observed at 5 and 10 years were 91% and 85%, respectively. These figures are similar to those published in other developed countries, as well as in the CONCORD-3 study and REDECAN [10, 33, 43, 45, 46]. However, as with the incidence data, the survival rate could be biased due to PSA-related overdiagnosis. In fact, this is one of the main limitations of this study, given that there are no PSA, Gleason or staging determinations at the time of diagnosis, nor data on the treatment received that allow us to analyze the role these two factors play in variations in incidence and survival. A further limitation of this study is that the mortality analysis was based solely on the information provided on death registries, and may therefore be underestimated. Finally, the results of this study cannot be extrapolated to other geographical populations given that they could have different environmental characteristics.

All of the above being said, this study presents contemporary population data on one of the most incident cancers in the population, with a trend analysis that highlights the progressive improvements that have been made in diagnosis and treatment of this disease. It is important to note that in their guidelines -the former from 2016 and the latter from 2018- the European Association of Urology and the American Cancer Society recommend that men not be subjected to PSA testing without advising on the risks and benefits, and with an agreement to share decision-making [8, 12]. Finally, more population-level studies are needed that include the staging of the disease at the time of diagnosis, as well as more clinical information and patient comorbidity that allow epidemiological data to be adjusted to the specific characteristics of the population.

### Supplementary Information

**Additional file 1:** **Table S1.** Age of diagnosis, crude and age-standardized incidence rates for PCa in Girona, 1994-2018. **Table S2.** Age of death, crude and age-standardized mortality rates for PCa in Girona, 1994-2018. **Figure S1.** Age-specific incidence and mortality rates for PCa in Girona, 1994-2018. **Table S3.** Five-year observed and net survival of PCa by age and period of diagnosis. **Table S4.** Ten-year observed and net survival of PCa according age and period of diagnosis. **Figure S2.** PSA requests in Girona, 2006-2018.

## Data Availability

The datasets generated for this study are available from the corresponding author upon request.
