# Supplementary material for: Significantly reduced incidence and improved survival from prostate cancer over 25 years
Source: BMC Public Health. 2023 Dec 21;23:2552. doi: 10.1186/s12889-023-17440-7 (PMC10734155; doi:10.1186/s12889-023-17440-7)
Supplement: Supplementary file 1 — Additional file 1: Table S1. Age of diagnosis, crude and age-standardized incidence rates for PCa in Girona, 1994-2018. Table S2. Age of death, crude and age-standardized mortality rates for PCa in Girona, 1994-2018. Figure S1. Age-specific incidence and mortality rates for PCa in Girona, 1994-2018. Table S3. Five-year observed and net survival of PCa by age and period of diagnosis. Table S4. Ten-year observed and net survival of PCa according age and period of diagnosis. Figure S2. PSA requests in Girona, 2006-2018. [file 12889_2023_17440_MOESM1_ESM.docx]

**Supplementary material**

Supplementary Table 1: Age of diagnosis, crude and age-standardized incidence rates for PCa in Girona, 1994-2018.

| **Year** | **N** | **Mean (± SD)** | **CR (95%CI)** | **ASRE (95%CI)** |
| --- | --- | --- | --- | --- |
| **1994** | 196 | 74.95 (7.64) | 75.60 (65.01 – 86.18) | 110.26 (94.85 − 127.86) |
| **1995** | 212 | 73.08 (8.45) | 81.24 (70.31 – 92.18) | 112.53 (97.42 − 129.71) |
| **1996** | 227 | 73.59 (8.93) | 86.58 (75.32 – 97.85) | 120.85 (105.09 – 138.66) |
| **1997** | 296 | 73.68 (8.22) | 112.26 (99.47 – 125.04) | 101.49 (87.40 – 117.54) |
| **1998** | 319 | 73.07 (8.14) | 119.84 (106.69 – 132.99) | 155.53 (138.53 − 174.38) |
| **1999** | 343 | 72.17 (8.16) | 127.40 (113.92 – 140.88) | 159.79 (143.05 – 178.76) |
| **2000** | 387 | 72.79 (8.70) | 141.44 (127.35 – 155.53) | 181.38 (163.43 − 201.05) |
| **2001** | 367 | 72.52 (9.02) | 130.61 (117.25 – 143.97) | 166.69 (149.75 − 185.30) |
| **2002** | 406 | 71.84 (8.65) | 139.58 (126.01 – 153.16) | 177.59 (160.51 − 196.26) |
| **2003** | 455 | 71.85 (8.80) | 150.42 (136.60 – 164.24) | 195.67 (177.85 − 215.04) |
| **2004** | 452 | 71.88 (8.88) | 143.04 (129.85 −156.23) | 188.84 (171.61 – 207.59) |
| **2005** | 481 | 71.98 (9.25) | 145.62 (132.61 – 158.64) | 193.84 (176.68 − 212.45) |
| **2006** | 454 | 71.35 (9.24) | 131.82 (119.70 – 143.95) | 180.66 (164.22 − 198.51) |
| **2007** | 448 | 71.35 (9.06) | 124.93 (113.36 −136.50) | 173.13 (157.30 − 190.30) |
| **2008** | 424 | 71.70 (9.42) | 114.57 (103.67 – 125.48) | 159.7 (144.71 − 175.96) |
| **2009** | 453 | 71.11 (9.55) | 120.51 (109.41 – 131.60) | 166.12 (151.04 − 182.41) |
| **2010** | 467 | 70.47 (9.72) | 123.56 (112.36 – 134.77) | 166.66 (151.74 − 182.74) |
| **2011** | 479 | 70.84 (9.74) | 126.34 (115.02 – 137.65) | 166.29 (151.59 – 182.10) |
| **2012** | 444 | 69.85 (9.96) | 117.67 (106.72 – 128.61) | 151.3 (137.41 – 166.31) |
| **2013** | 431 | 69.95 (9.20) | 115.15 (104.28 – 126.02) | 146.35 (132.72 – 161.09) |
| **2014** | 386 | 69.63 (9.94) | 104.02 (93.64 – 114.39) | 128.26 (115.66 – 141.91) |
| **2015** | 383 | 70.21 (8.58) | 103.65 (93.27 – 114.03) | 126.85 (114.35 – 140.41) |
| **2016** | 437 | 70.56 (8.87) | 118.07 (107.00 – 129.15) | 140.89 (127.86 – 154.96) |
| **2017** | 444 | 70.85 (9.04) | 119.44 (108.33 – 130.55) | 142.56 (129.47 – 156.69) |
| **2018** | 455 | 70.80 (8.80) | 121.42 (110.26 – 132.58) | 141.67 (128.85 – 155.51) |

ASRE, age-standardized rate (European), CI: confidence interval, CR: crude rate, SD: standard deviation

Supplementary Table 2: Age of death, crude and age-standardized mortality rates for PCa in Girona, 1994-2018.

| **Year** | **N** | **Mean (±SD)** | **CMR (95%CI)** | **ASMRE (95%CI)** |
| --- | --- | --- | --- | --- |
| **1994** | 87 | 79.17 (7.27) | 33.55 (26.50 – 40.61) | 54.44 (43.19 – 68.06) |
| **1995** | 82 | 78.64 (7.72) | 31.42 (24.62 – 38.23) | 48.61 (38.25 – 61.24) |
| **1996** | 86 | 79.28 (8.01) | 32.80 (25.87 – 39.74) | 52.07 (41.28 – 65.08) |
| **1997** | 73 | 79.01 (8.7) | 27.68 (21.33 – 34.04) | 42.96 (33.30 – 54.79) |
| **1998** | 98 | 79.39 (7.92) | 36.82 (29.53 – 44.10) | 55.29 (44.51 – 68.14) |
| **1999** | 83 | 79.43 (7.61) | 30.83 (24.20 – 37.46) | 44.62 (33.30 – 54.79) |
| **2000** | 106 | 80.05 (8.49) | 38.74 (31.37 – 46.12) | 57.74 (46.99 –70.41) |
| **2001** | 88 | 81.00 (8.29) | 31.32 (24.77 – 37.86) | 46.80 (37.29 – 58.20) |
| **2002** | 101 | 79.59 (7.94) | 34.72 (27.95 – 41.50) | 49.39 (39.99 – 60.55) |
| **2003** | 85 | 81.08 (7.27) | 28.10 (22.13 – 34.07) | 42.08 (33.38 – 52.54) |
| **2004** | 100 | 79.80 (9.82) | 31.65 (25.44 – 37.85) | 47.59 (38.46 – 58.42) |
| **2005** | 101 | 79.93 (8.03) | 30.58 (24.61 – 36.54) | 45.94 (37.18 – 56.32) |
| **2006** | 75 | 81.27 (8.76) | 21.78 (16.85 – 26.71) | 33.92 (26.49 – 42.93) |
| **2007** | 83 | 80.89 (8.05) | 23.15 (18.17 – 28.12) | 34.86 (27.63 – 43.55) |
| **2008** | 86 | 81.88 (8.18) | 23.24 (18.33 – 28.15) | 35.33 (28.14 – 43.91) |
| **2009** | 81 | 82.10 (8.03) | 21.55 (16.85 – 26.24) | 32.76 (25.93 – 40.92) |
| **2010** | 95 | 81.44 (8.97) | 25.14 (20.08 – 30.19) | 36.85 (29.71 – 45.24) |
| **2011** | 103 | 80.74 (8.68) | 27.17 (21.92 – 32.41) | 37.55 (30.59 – 45.70) |
| **2012** | 73 | 81.45 (8.74) | 19.35 (14.91 – 23.78) | 26.49 (20.72 – 33.45) |
| **2013** | 90 | 81.79 (8.49) | 24.05 (19.08 – 29.01) | 32.01 (25.70 – 39.49) |
| **2014** | 99 | 82.19 (8.49) | 26.68 (21.42 – 31.93) | 34.25 (27.79 – 41.80) |
| **2015** | 104 | 81.72 (9.26) | 28.14 (22.74 – 33.55) | 35.13 (28.66 – 42.70) |
| **2016** | 105 | 80.55 (10.61) | 28.37 (22.94 – 33.80) | 34.77 (28.40 – 42.23) |
| **2017** | 81 | 81.95 (9.23) | 21.79 (17.04 – 26.53) | 26.39 (20.92 – 32.94) |
| **2018** | 89 | 82.24 (9.14) | 23.71 (18.82 – 28.68) | 28.95 (23.22 – 35.74) |

ASMRE, age-standardized mortality rate (European), CI: confidence interval, CMR: crude mortality rate, SD: standard deviation

Supplementary Figure 1. Age-specific incidence and mortality rates for PCa in Girona, 1994-2018.

Supplementary Table 3. Five-year observed and net survival of PCa by age and period of diagnosis.

|  | **1994-1998** | **1999-2003** | **2004-2008** | **2009-2013** | **2014-2018*** |
| --- | --- | --- | --- | --- | --- |
|  | **5-year OS (95%CI)** | | | | |
| **Age (years)**  <55  55-64  65-74  75-84  ≥85 | 70.2 (45.3-85.4)  76.8 (68.9-83.0)  66.1 (61.8-70.0)  47.4 (42.5-52.1)  19.6 (12.6-27.8) | 94.0 (82.5-98.0)  89.7 (85.7-92.7)  76.6 (73.5-79.4)  57.5 (53.5-61.3)  21.1 (14.0-29.2) | 96.1 (88.5-98.7)  92.5 (89.6-94.7)  82.3 (79.6-84.7)  60.0 (56.2-63.6)  30.8 (23.2-38.8) | 89.6 (81.5-94.2)  92.1 (89.5-94.0)  86.3 (83.8-88.5)  64.9 (61.0-68.5)  27.6 (20.8-34.7) | 92.6 (88.1-95.5)  91.2 (89.4-92.8)  86.1 (84.5-87.7)  64.6 (62.0-67.1)  27.3 (22.4-32.4) |
| **Overall** | **56.9 (54.0-59.7)** | **69.7 (67.6-71.7)** | **74.7 (72.9-76.5)** | **77.9 (76.1-79.5)** | **77.8 (76.6-79.0)** |
|  | **5-year NS (95%CI)** | | | | |
| **Age (years)**  <55  55-64  65-74  75-84  ≥85 | 72.3 (45.7-87.4)  82.0 (73.0-88.2)  77.7 (72.4-82.1)  70.3 (62.4-76.9)  47.4 (27.6-64.9) | 96.6 (77.0-99.5)  95.1 (89.8-97.7)  88.6 (84.6-91.5)  81.8 (75.2-86.7)  47.2 (27.7-64.4) | 98.6 (72.8-99.9)  97.7 (92.9-99.2)  93.6 (89.9-95.9)  83.3 (77.3-87.9)  71.8 (47.9-86.2) | 91.4 (82.5-95.9)  96.9 (93.4-98.5)  96.3 (92.5-98.2)  87.0 (81.1-91.5)  60.6 (43.4-74.0) | 94.6 (89.5-97.3)  96.0 (93.8-97.5)  96.3 (94.0-97.7)  86.9 (82.8-90.0)  60.3 (48.0-70.5) |
| **Overall** | **72.9 (68.8-76.6)** | **85.2 (82.1-87.8)** | **90.0 (87.3-92.2)** | **91.2 (88.7-93.2)** | **91.3 (89.6-92.7)** |

* period approach

Supplementary Table 4. Ten-year observed and net survival of PCa according age and period of diagnosis.

|  | **1994-1998** | **1999-2003** | **2004-2008** | **2009-2013** |
| --- | --- | --- | --- | --- |
|  | **10-year OS (95%CI)** | | | |
| **Age (years)**  <55  55-64  65-74  75-84  ≥85 | 55.1 (31.5-73.6)  58.0 (49.3-65.7)  48.2 (43.8-52.5)  20.2 (16.4-24.2)  2.9 (0.8-7.6) | 84.0 (70.5-91.7)  81.5 (76.6-85.4)  57.0 (53.5-60.4)  27.1 (23.6-30.6)  2.8 (0.7-7.2) | 93.6 (85.3-97.3)  82.5 (78.5-85.8)  62.7 (59.3-65.8)  29.8 (26.4-33.3)  6.8 (3.3-11.9) | 83.8 (69.4-91.8)  80.4 (75.6-84.4)  68.4 (64.0-72.4)  38.2 (33.6-42.8)  4.8 (1.8-10.0) |
| **Overall** | **35.8 (33.1-38.5)** | **48.7 (46.4-50.9)** | **54.0 (51.9-56.1)** | **59.0 (56.5-61.5)** |
|  | **10-year NS (95%CI)** | | | |
| **Age (years)**  <55  55-64  65-74  75-84  ≥85 | 59.1 (32.7-78.1)  67.9 (57.1-76.5)  72.2 (65.0-78.1)  51.1 (40.3-61.6)  20.8 (3.7-47.4) | 89.7 (71.9-96.5)  93.6 (86.1-97.1)  81.4 (75.8-85.9)  63.6 (54.0-71.7)  20.2 (3.6-46.2) | 99.6 (0-100)  94.0 (88.2-97.0)  86.2 (80.9-90.1)  67.7 (58.5-75.3)  43.5 (14.2-70.0) | 88.6 (71.6-95.7)  91.5 (85.2-95.1)  91.2 (83.8-95.3)  83.6 (68.9-91.7)  29.6 (7.6-56.2) |
| **Overall** | **59.8 (53.7-65.4)** | **74.2 (69.5-78.3)** | **79.9 (75.4-83.6)** | **85.1 (79.9-89.0)** |

Supplementary Figure 2. PSA requests in Girona, 2006-2018
